# Genetic Characterization and Zoonotic Potential of Highly Pathogenic Avian Influenza Virus A(H5N6/H5N5), Germany, 2017–2018

**DOI:** 10.3201/eid2510.181931

**Published:** 2019-10

**Authors:** Anne Pohlmann, Donata Hoffmann, Christian Grund, Susanne Koethe, Daniela Hüssy, Simone M. Meier, Jacqueline King, Jan Schinköthe, Reiner Ulrich, Timm Harder, Martin Beer

**Affiliations:** Friedrich-Loeffler-Institut, Greifswald-Insel Riems, Germany (A. Pohlmann, D. Hoffmann, C. Grund, S. Koethe, J. King, J. Schinköthe, R. Ulrich, T. Harder, M. Beer);; Institute of Virology and Immunology, Mittelhäusern, Switzerland (D. Hüssy);; Vetsuisse Faculty Zurich, Zurich, Switzerland (S.M. Meier)

**Keywords:** highly pathogenic avian influenza, HPAI, H5N6, H5N5, H5N8, H5Nx, zoonotic potential, epizootic, reassortment, zoonoses, clade 2.3.4.4b, Germany, ferret model, influenza, viruses, respiratory infections

## Abstract

We genetically characterized highly pathogenic avian influenza virus A(H5N6) clade 2.3.4.4b isolates found in Germany in 2017–2018 and assessed pathogenicity of representative H5N5 and H5N6 viruses in ferrets. These viruses had low pathogenicity; however, continued characterization of related isolates is warranted because of their high potential for reassortment.

During winter 2016–17, outbreaks of highly pathogenic avian influenza (HPAI) virus A(H5N8) clade 2.3.4.4b caused substantial losses in wild water birds and domestic poultry across Europe (*1*–*4*). This virus is related to strains from China and Mongolia and has a high potential for reassortment (*4*–*6*). Genetic and temporal analysis of these isolates revealed multiple reassortant events, indicating multiple independent entries into Europe; the outbreaks in Germany were dominated by 5 independent reassortant groups of HPAI virus H5N8 (*5*). Several outbreaks of HPAI virus H5Nx strains in wild birds confirmed the continued presence of H5 clade 2.3.4.4b in Europe well into the summer of 2017. This virus’s high tendency to reassort raised concerns that further reassorted strains could dominate in HPAI outbreaks in Europe or become enzootic in wild bird populations in the future. In this study, we set out to characterize related reassortant viruses of subtype H5N6 or H5N5 isolated in Germany during 2017–2018 and delineate their zoonotic potential in ferrets.

## The Study

Starting in November 2017, H5 HPAI viruses, classified as clade 2.3.4.4b according to their hemagglutinin (HA) segments, carrying N6 segments were detected in the Netherlands (*7*), United Kingdom, Switzerland, and Germany (*8*). We used samples mostly from the outbreaks in Germany collected during December 2017–August 2018 (Appendix Table 1). We sequenced (Appendix) and analyzed these viruses and found they carried a neuraminidase (NA) segment of subtype N6 with a high similarity to low pathogenicity avian influenza (LPAI) viruses identified in Asia during 2015–2017 (Appendix Table 2).

According to a full-genome analysis, these H5N6 viruses represent 2 mosaic reassortants of HPAI virus H5N8 found in Europe during the epizootic of 2016–17 (Figure, panel A). Reassortant group I shares all but the NA segment with viruses from the epizootic of 2016–17 (Appendix Figure 1), and because of distinct homologies in the HA, matrix, and nonstructural protein gene segments (Appendix Figure 1), these viruses were further divisible into 3 subgroups, which we designated Gre-02-17-N6, Tai-12-17-N6, and Kor-12-17-N6 (Figure, panel A). The divergence within this reassortant group might have been caused by genetic drift and would be in line with their temporal and geographic patterns of occurrence (Figure, panel B). In contrast, reassortant group II (designated Ger-12-17-N6; Figure) comprises a more homogeneous group of H5N6 viruses from Western and Central Europe. Reassortant group II is genetically distinguishable from reassortant group I by separate clustering of the polymerase acidic (PA) and polymerase basic 2 (PB2) genes (Appendix Figure 1). Group II viruses were detected in Germany during December 2017–August 2018. Their PA segment is similar to that of the HPAI virus A(H5N8) found in the Netherlands in November and December 2016, and their PB2 segment is similar to that of LPAI viruses in Europe and, to a lesser extent, HPAI H5N5 and H5N8 2.3.4.4b isolates from the epizootic of 2016–17 (Appendix Figure 1). This finding underscores the ability of HPAI virus clade 2.3.4.4b from the epizootic of 2016–17 to frequently reassort, probably empowered by its genome constellation, especially its HA segment.

**Figure:**
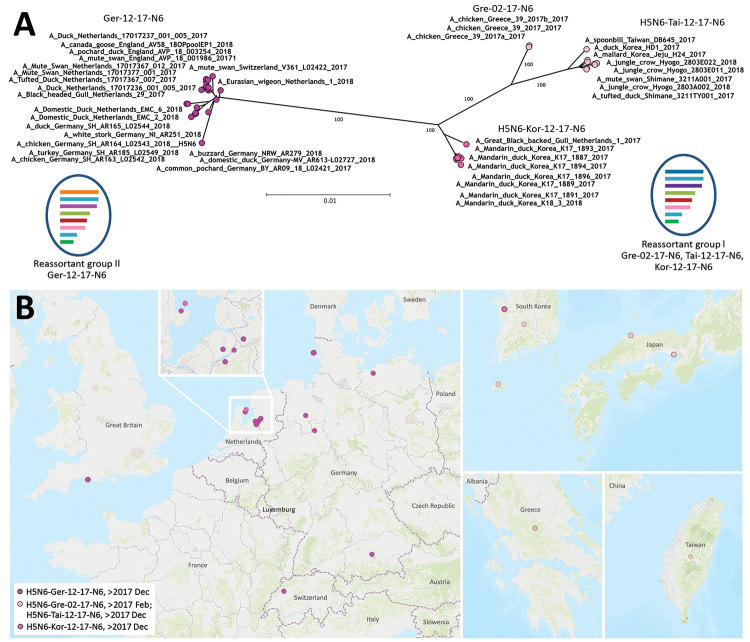
Phylogenetic clustering and geographic distribution of highly pathogenic avian influenza A(H5N6) viruses, Europe, 2017–2018. A) Supernetwork generated by using maximum-likelihood trees of influenza virus full genomes with RAxML (https://cme.h-its.org/exelixis/web/software/raxml/index.html) and 1,000 bootstrap iterations followed by network analysis with SplitsTree4 (http://ab.inf.uni-tuebingen.de/software/splitstree4). Reassortant viruses are grouped according to their phylogenetic results. Scale bar indicates nucleotide substitutions per site. The mosaic genome structure of reassortant groups I and II is also provided. Gene segment descriptions are given in Appendix Figure). B) Geographic locations of cluster isolates. Inset of cluster in the Netherlands is provided for easier visualization. L, Luxembourg.

H5N6 viruses of clades 2.3.4.4c and 2.3.4.4d and an H5 virus of clade 2.3.4.4b (A/Fujian-Sanyuan/21099/2017) have been reported in cases of human influenza; thus, concerns have been raised about these viruses’ zoonotic potential (*9*). Several clade 2.3.4.4b H5 HPAI viruses isolated in South Korea (Appendix Table 3) have already been evaluated in multiple animal models and showed no zoonotic propensity in ferrets (*10*,*11*). These results concur with our previous analysis of cluster 2.3.4.4b HPAI virus H5N8 from Germany (A/tufted_duck_Germany/AR8444/2016) in human lung explants and in ferrets (*12*).

We extended the zoonotic risk assessment of these viruses by using a reassortant group II HPAI H5N6 virus (AR09/18, A/common_pochard/Germany-BY/AR09-18-L02421/2017). For comparison, we included a related reassortant HPAI H5N5 clade 2.3.4.4b virus (AR425/17, A/turkey/Germany-SH/R425/2017) with 3 genes, NA (Appendix Table 2), polymerase basic 1, and nucleoprotein, related to LPAI viruses from different countries and 4 genes, PB2, PA, matrix, and nonstructural protein, related to HPAI viruses isolated during the epizootic of 2016–17 (*5*).

We inoculated 10 ferrets intranasally with either the H5N6 or H5N5 virus (Appendix). None of the animals displayed any respiratory signs; the only change observed was a minor, short-lived increase in body temperature. Only 1 of the 5 ferrets inoculated with H5N5 exhibited body temperatures >40°C for 3 consecutive days (5–7 days postinfection [dpi]). This particular animal also exhibited a mild gait disorder at 5 dpi, and because these atactic movements persisted (a sign qualifying for termination), the ferret was euthanized at 7 dpi. The viral RNA loads in the nasal washings of animals inoculated with H5N5 and H5N6 were low up through 7 dpi (Table), and RNA excretion ceased thereafter. However, at 7 dpi, the H5N5-inoculated ferret showing mild ataxia displayed a peak of 100 copies/µL of extracted RNA (input volume 100 µL) in the nasal washing fluid (Table).

**Table:** Viral RNA loads in nasal washing samples from ferrets infected with highly pathogenic avian influenza A(H5N6/H5N5) clade 2.3.4.4b virus isolates from Germany, 2017–2018, and seroconversion in study assessing virus zoonotic risk*

Group	Day postinfection, viral RNA load, genome copies/µL						Seroconversion†
	0	1	3	5	7	9	
Controls	–	–	–	–	–	–	–
H5N5	–	0.2	0.2	0.1	100.6	ND‡	+/−§
	–	1.2	2.8	0.1	–	–	+
	–	1.9	0.9	2.9	–	–	+
	–	1.2	–	0.1	–	–	+
	–	0.1	–	8.2	0.9	–	+
H5N6	–	–	0.8	1.3	1.3	–	+
	–	0.2	1.9	13.8	3.2	–	+
	–	0.2	–	0.2	0.2	–	+
	–	–	–	–	–	–	+
	–	0	1.3	0.2	4.2	–	+

*ND, not done; –, negative. †Seroconversion measured with sensitive nucleoprotein ELISA (ID Screen Influenza A Antibody Competition ELISA Kit; ID.Vet, https://www.id-vet.com) with day 14 or 7 serum sample, depending on day of animal sacrifice, and compared with preinoculation serum sample. ‡Sacrificed day 7 because animal displayed neurologic symptoms. §Reaction interpreted as reactive but not positive.

Nucleoprotein antibody–specific seroconversion (Table) was detected in all inoculated ferrets surviving until euthanasia at 14 dpi. The serum sample of the atactic animal euthanized at 7 dpi scored reactive but not positive.

We dissected all euthanized animals and analyzed spleens, tracheas, lungs, conchae, cerebellum, and cerebrum for viral genome loads, as described previously (*12*). All organ samples taken at 14 dpi were negative; however, the cerebrum, trachea, and nasal concha of the single animal exhibiting disease euthanized at 7 dpi had a low viral load of 7–25 copies/µL of extracted RNA from ≈2 mm^3^ tissue material homogenized in 1 mL of medium (input volume 100 µL).

Histopathologic workup of the sick ferret revealed mild, subacute necrotizing rhinitis; moderate, oligofocal, subacute necrotizing bronchointerstitial pneumonia; moderate, multifocal necrotizing hepatitis; severe necrotizing salpingitis; and the focal-to-multifocal intralesional presence of influenza virus matrix protein (Appendix Figure 2) consistent with systemic virus spread. Only 1 of 4 of the H5N5-infected ferrets and 2 of 5 of the H5N6-infected ferrets necropsied at 14 dpi revealed inflammatory lung lesions, yet all were negative for matrix protein by immunohistochemical staining (Appendix Table 4). Considering the low morbidity rate (10%), these H5 viruses have a mild pathogenic potential in the ferret model compared with other HPAI viruses (*13*).

## Conclusions

The genetic makeup of HPAI H5 clade 2.3.4.4b viruses fosters reassortment, which can expand their evolutionary capacity. Segment reassortment bears a concomitant danger of the emergence of strains that are more pathogenic or zoonotic or that have a higher potential to evolve to propagate in avian hosts with different migratory behaviors. H5N6 and H5N5 viruses of this clade have been continuously present in Europe since 2017, necessitating continuous surveillance and virus characterization. Our study excludes the possibility of enhanced zoonotic potential for the analyzed H5N5 and H5N6 2.3.4.4b clade viruses. Nonetheless, existing reports of clade 2.3.4.4c HPAI H5N6 virus infections in mammals and clade 2.3.4.4b-2.3.4.4d virus co-infections in humans indicate a continued risk for zoonotic events with H5Nx reassortants (*9*). Continued surveillance and characterization of these viruses is crucial to reduce the risk for outbreaks with burgeoning HPAI isolates of the goose/Guangdong lineage.

Appendix More information about genetic characterization and zoonotic potential of highly pathogenic avian influenza virus A(H5N6/H5N5), Germany, 2017–2018.
