# Supplementary material for: Genetic Characterization and Zoonotic Potential of Highly Pathogenic Avian Influenza Virus A(H5N6/H5N5), Germany, 2017–2018
Source: Emerg Infect Dis. 2019 Oct;25(10):1973–6. doi: 10.3201/eid2510.181931 (PMC6759249; doi:10.3201/eid2510.181931)
Supplement: Appendix — More information about genetic characterization and zoonotic potential of highly pathogenic avian influenza virus A(H5N6/H5N5), Germany, 2017–2018. [file 18-1931-Techapp-s1.pdf]

Article DOI: https://doi.org/10.3201/eid2510.181931

Genetic Characterization and Zoonotic Potential of Highly Pathogenic Avian Influenza Virus A(H5N6/H5N5), Germany, 2017–2018

Appendix

Methods

Sequencing and Data Evaluation

RNA of influenza-positive samples was extracted by using Trizol LS (ThermoFisher Scientific, Waltham, USA) and QIAamp Viral RNA Mini Kit (QIAGEN, Hilden, Germany). Influenza genome segments were amplified with specific primers by using Invitrogen Superscript III One-Step RT-PCR with Platinium Taq (ThermoFisher Scientific, Waltham, USA). The reverse transcription PCR (RT-PCR) amplicons were sequenced by Sanger or next generation sequencing (NGS) as previously described (*1*,*2*). For NGS, fragmentation of the RT-PCR amplicons was done with a Covaris M220 Ultrasonicator (Covaris Ltd, Brighton, UK) applying a target size of 500 bp. The sonicated cDNA was used for library preparation by using IonTorrent Ion Xpress Barcode Adapters and GeneRead DNA Library L Core Kit (QIAGEN). Size exclusion of the library was done with AMPure XP Magnetic Beads (Beckman Coulter, Fullerton, USA). The libraries were quality checked by using High Sensitivity DNA Chips and reagents on a Bioanalyzer 2100 (Agilent Technologies, Böblingen, Germany), quantized via quantitative PCR with KAPA Library Quantification Kit Ion Torrent (Roche, Mannheim, Germany), and sequenced on an IonTorrent PGM or S5 XL (Thermo Scientific), respectively. Raw sequence data were quality-trimmed and screened for adapter and primer contamination. Consensus sequences were generated with an iterative mapping approach by using Bowtie2 (v. 2.3.0) (*3*) in the Geneious software suite (v. 10.2.3; Biomatters, Auckland, New Zealand). Geneious software suite was also used for quality check and automatic annotation of the sequences. Strain details and epidemiologic information of the viruses sequenced in this study

are given in Appendix Table 1. For network analysis, virus sequences of German high pathogenicity avian influenza (HPAI) H5N6 viruses sequenced in this study (Appendix Table 1) were aligned by segment together with similar sequences from HPAI H5N6 strains aligned by using MAFFT (*4*) (scoring matrix 200PAM/k = 2, gap penalty 1.53, two iterative refinement cycles) receiving 8 alignments, 1 for each segment. Phylogenetic analyzes of these alignments were done by using RAxML (*5*) with general time-reversible plus gamma as the substitution model applying 1,000 bootstrap replicates, resulting in 8 bootstrap-supported phylogenetic trees. All 8 trees were imported into SplitsTree4 for network generation (SuperNetwork, using mean edge weighting, including Z-rule, using Equal Angle for weighted splits transformation, Convex Hull and greedily compatibility applied) (*6*). Maps were created by using ArcGIS Online (www.arcgis.com). Sequences for comparison were retrieved from the Influenza Research Database (www.fludb.org) and EpiFlu Database (www.gisaid.org). We acknowledge the laboratories for providing sequence information via EpiFlu listed in Appendix Table 5. Consensus sequences were published in the EpiFlu Database under accession EPI_ISL_291109, EPI_ISL_291110, EPI_ISL_305453, EPI_ISL_305454, EPI_ISL_305455, EPI_ISL_306989, EPI_ISL_313226, EPI_ISL_313227, and EPI_ISL_322179.

Dataset Selection

The sequence dataset for the phylogeny were selected to include representatives of related strains from the epizootic 2016–2017 and from European and Asian H5N6 outbreaks 2017–2018. In addition, HPAI strains used in animal trials or isolated from humans and, for PB2 phylogeny, similar sequences from low pathogenicity avian influenza viruses are included.

Animal Trial

We tested a HPAIV H5N6 virus (AR09/18; A/common_pochard/Germany-BY/AR09–18-L02421/2017) together with a different reassortant HPAIV H5N5 (AR425/17; A/turkey/Germany-SH/R425/2017) in a ferret inoculation model. The animal experiments gained legal governmental approval through the ethics committee of the State Office of Agriculture, Food Safety, and Fishery in Mecklenburg-Vorpommern (LALLF M-V: LVL MV/TSD/ 7221.3–1.1–023/13). All procedures were carried out in approved biosafety level 3 facilities. We inoculated 5 ferrets (including additional control animals which were not inoculated) per virus subtype intranasally using $10^{5.5}$ 50% tissue culture infectious dose ($TCID_{50}$) per animal of H5N6 AR09/18 (total 75 µL) or 10^{6} $TCID_{50}$ per animal of H5N5 AR425/17 (total 50 µL). Nasal washes

were collected every other day from all ferrets to measure virus excretion by applying 1 mL phosphate-buffered saline into each nostril. Analyses of viral RNA load from nasal washes and organ samples were performed exactly as described (*7*). Ferret serum samples taken preinoculation and at 14 days post infection were heat-inactivated at 56°C for 30 min and analyzed by means of a commercial ELISA for the presence of antibodies against influenza A Virus nucleoprotein (ID Screen Influenza A Antibody Competition ELISA Kit, ID-vet, Montpellier, France) according to the manufacturer´s instructions. Hemagglutination inhibition assays against the homologous H5N5 and H5N6 antigens were performed according to standard protocols (Commission, E. 2006/437/EC: Commission Decision of 4 August 2006 approving a Diagnostic Manual for avian influenza as provided for in Council Directive 2005/94/EC. Report No. ISSN 1725–2555, 16 [2006]).

Strain details and epidemiologic information of the viruses sequenced in this study are summarized in Appendix Table 1. Phylogenetic analyzes of segments were done with similar sequences as described above. The results are given in Appendix Figure 1.

References

1. Pohlmann A, Starick E, Grund C, Höper D, Strebelow G, Globig A, et al. Swarm incursions of reassortants of highly pathogenic avian influenza virus strains H5N8 and H5N5, clade 2.3.4.4b, Germany, winter 2016/17. Sci Rep. 2018;8:15. PubMed https://doi.org/10.1038/s41598-017-16936-8

2. Pohlmann A, Starick E, Harder T, Grund C, Höper D, Globig A, et al. Outbreaks among wild birds and domestic poultry caused by reassorted influenza A(H5N8) clade 2.3.4.4 viruses, Germany, 2016. Emerg Infect Dis. 2017;23:633–6. PubMed https://doi.org/10.3201/eid2304.161949

3. Langmead B, Salzberg SL. Fast gapped-read alignment with Bowtie 2. Nat Methods. 2012;9:357–9. PubMed https://doi.org/10.1038/nmeth.1923

4. Katoh K, Standley DM. MAFFT multiple sequence alignment software version 7: improvements in performance and usability. Mol Biol Evol. 2013;30:772–80. PubMed https://doi.org/10.1093/molbev/mst010

5. Stamatakis A. RAxML version 8: a tool for phylogenetic analysis and post-analysis of large phylogenies. Bioinformatics. 2014;30:1312–3. PubMed https://doi.org/10.1093/bioinformatics/btu033

6. Huson DH, Bryant D. Application of phylogenetic networks in evolutionary studies. Mol Biol Evol. 2006;23:254–67. PubMed https://doi.org/10.1093/molbev/msj030

7. Grund C, Hoffmann D, Ulrich R, Naguib M, Schinköthe J, Hoffmann B, et al. A novel European H5N8 influenza A virus has increased virulence in ducks but low zoonotic potential. Emerg Microbes Infect. 2018;7:132. PubMed https://doi.org/10.1038/s41426-018-0130-1

8. Centers for Disease Control and Prevention. H5N1 genetic changes inventory: a tool for influenza surveillance and preparedness. 2012 Jun 26 [cited 2018 Aug 14]. https://www.cdc.gov/flu/pdf/avianflu/h5n1-inventory.pdf

9. World Health Organization. Antigenic and genetic characteristics of zoonotic influenza viruses and development of candidate vaccine viruses developed for pandemic preparedness. 2018 Feb [cited 2018 Feb 22]. http://www.who.int/entity/influenza/vaccines/virus/201802_zoonotic_vaccinevirusupdate.pdf?ua=1

10. Kwon HI, Kim EH, Kim YI, Park SJ, Si YJ, Lee IW, et al. Comparison of the pathogenic potential of highly pathogenic avian influenza (HPAI) H5N6, and H5N8 viruses isolated in South Korea during the 2016-2017 winter season. Emerg Microbes Infect. 2018;7:29. PubMed https://doi.org/10.1038/s41426-018-0029-x

11. Noh JY, Lee DH, Yuk SS, Kwon JH, Tseren-Ochir EO, Hong WT, et al. Limited pathogenicity and transmissibility of Korean highly pathogenic avian influenza H5N6 clade 2.3.4.4 in ferrets. Transbound Emerg Dis. 2018;65:923–6. PubMed https://doi.org/10.1111/tbed.12869

Appendix Table 1. Strain details and epidemiologic information of the viruses sequenced in this study

Name	Subtype	Country	Federal state	Host	Collection date	Reference
A/tufted_duck/Germany/AR8444/2016*	H5N8	Germany	Schleswig-Holstein	*Aythya fuligula*	2016 Nov 7	(*2*)
A/turkey/Germany-SH/R425/2017*	H5N5	Germany	Schleswig-Holstein	Domestic turkey	2017 Jan 22	(*1*)
A/common_pochard/Germany-BY/AR09–18-L02421/2017*	H5N6	Germany	Bavaria	*Aythya ferina*	2017 Dec 28	This study
A/mute_swan/Switzerland/V361-L02422/2017	H5N6	Switzerland	Kanton Bern, Erlach	*Cygnus olor*	2017 Dec 18	This study
A/white_stork/Germany-NI/AR251/2018	H5N6	Germany	Lower Saxony	*Ciconia ciconia*	2018 Apr	This study
A/buzzard/Germany-NRW/AR279/2018	H5N6	Germany	North Rhine-Westphalia	*Buteo*	2018 May	This study
A/chicken/Germany-SH/AR163-L02542/2018	H5N6	Germany	Schleswig-Holstein	Domestic chicken	2018 Mar 19	This study
A/chicken/Germany-SH/AR164-L02543/2018	H5N6	Germany	Schleswig-Holstein	Domestic chicken	2018 Mar 19	This study
A/duck/Germany-SH/AR165-L02544/2018	H5N6	Germany	Schleswig-Holstein	Domestic duck	2018 Mar 19	This study
A/turkey/Germany-SH/AR185-L02549/2018	H5N6	Germany	Schleswig-Holstein	Domestic turkey	2018 Mar 19	This study
A/domestic_duck/Germany-MV/AR613-L02727/2018	H5N6	Germany	Mecklenburg-Vorpommern	Domestic duck	2018 Aug 31	This study

*Used in animal trial.

Appendix Table 2. NA segment similarities (protein and nucleotide) for the N6 and N5 segment of virus A/common_pochard/Germany-BY/AR09–18-L02421/2017 (N6) and A/turkey/Germany-SH/R425/2017 (N5), respectively, compared with similar NA segments from low pathogenicity avian influenza viruses of different subtypes*

Segment	Accession no.	Name	Protein identity	Nucleotide identity
N6	MF694081	A/mallard duck/Georgia/9/2016 (A/H4N6)	457/470 (97%)	1411/1441 (97%)
	MF694113	A/mallard duck/Georgia/3/2016 (A/H4N6)	457/470 (97%)	1412/1441 (97%)
	MH135674	A/duck/Bangladesh/33676/2017 (A/H4N6)	452/470 (96%)	1351/1436 (94%)
	MH071489	A/duck/Bangladesh/24268/2015 (A/H10N6)	450/470 (95%)	1363/1446 (94%)
	LC121366	A/duck/Mongolia/405/2015 (A/H3N6)	450/470 (95%)	1329/1443 (92%)
	LC121262	A/duck/Mongolia/118/2015 (A/H4N6)	450/470 (95%)	1329/1443 (92%)
	KY635728	A/duck/Bangladesh/25891/2015 (A/H4N6)	450/470 (95%)	1356/1446 (93%)
	KY635782	A/duck/Bangladesh/26920/2015 (A/H3N6)	449/470 (95%)	1358/1447 (93%)
N5	KU9629324	A/common redshank/Singapore/F83–1/2015 (A/H9N5)	468/472 (99%)	1398/1418 (98%)
	KP285887	A/migratory duck/Jiangxi/31577/2013 (A/H10N5)	467/472 (98%)	1389/1418 (97%)
	EPI866833	A/duck/Aichi/231002/2016 (A/H6N5)	465/472 (98%)	1383/1423 (97%)
	EPI866951	A/duck/Fukui/181006/2015 (A/H12N5)	462/472 (97%)	1377/1423 (96%)
	KY635758	A/black-tailed godwit/Bangladesh/24734/2015 (A/H7N5)	463/472 (98%)	1386/1423 (97%)
	MF613702	A/shorebird/Delaware Bay/327/2016 (A/H10N5)	454/472 (96%)	1295/1422 (91%)
	MF613865	A/American black duck/Alberta/274/2016 (A/H10N5)	454/472 (96%)	1296/1422 (91%)
	CY240796	A/ruddy turnstone/New Jersey/UGAI16–1448/2016 (A/H10N5)	454/472 (96%)	1299/1427 (91%)

*Typical amino acid markers that are known to support replication in mammalian hosts, for example PB2 627K and PB2 701N (*8*), are not present in the tested viruses and in the first clade 2.3.4.4b human isolate FuSa21099/17. The receptor binding site in the HA segments of the analyzed viruses also indicated a preferred binding to α-2,3 sialic acids present in the avian respiratory tract. Furthermore, H5N8 AR8444/16 and H5N6 AR09/18 NS1 protein do not show a C-terminal PDZ binding motif, which is associated with increased virulence in mice (*8*). HA, hemagglutinin; NA, neuraminidase; PB2, polymerase basic protein 2.

Appendix Table 3. H5Nx-strains tested in the ferret animal model described in this study or in different animal models, and human H5Nx-strains, summarizing their abbreviation, names, and references for their evaluation of zoonotic potential with corresponding relevant genetic markers*

Abbreviation	Name	Subtype	HA recombination binding site	PB2 627	PB2 701	NS PBM	Reference
FuSa21099/17	A/Fujian-Sanyuan/21099/2017	H5N6	QVNGQRG	E	D	GSEV	(*9*)
EMW541/16	A/Environment/Korea/W541/2016	H5N6	QVNGQQG	E	D	ESEV	(*10*)
CTW555/17	A/Common Teal/Korea/W555/2017	H5N8	QVNGQRG	E	D	GSEV	(*10*)
MDK16/16	A/Mandarin duck/Korea/K16–187–3/2016	H5N6	QVNGQQG	E	D	ESEV	(*11*)
AR8444/16	A/tufted_duck_Germany/AR8444/2016	H5N8	QVNGQRG	E	D	†	(*7*)
AR425/17	A/turkey/Germany-SH/R425/2017	H5N5	QVNGQRG	E	D	GSEV	This study
AR09/18	A/common_pochard/Germany-BY/AR09–18-L02421/2017	H5N6	QVNGQRG	E	D	†	This study

*HA, hemagglutinin; NS, nonstructural protein; PB2, polymerase basic 2; PBM, PDZ binding motif.
†NS PBM not present. Truncated protein.

Appendix Table 4. Immunohistological evaluation of tested ferret tissue*

Group	Necropsy, dpi	R.E.	O.E.	Nasolacrimal duct	Sinus maxillaris	Tonsilla palatina	Lungs	Heart	Spleen	Liver	Jejunum	Pancreas	Colon	Kidney	Cerebrum	Cerebellum	M.O.	Other
Neg. control	14	0	ND	ND	ND	0	0	0	0	0	0	0	0	0	0	0	0	
Neg. control	14	0	0	0	ND	0	0	0	0	0	0	0	ND	0	0	0	0	
H5N5	7	0	2	0	0	ND	1	0	0	2	0	0	0	0	0	0	0	1 oral mucosa, 2 ovary, 3 salpinx
H5N5	14	0	0	0	0	0	0	0	0	0	0	0	ND	0	0	0	0	
H5N5	14	0	0	ND	ND	0	0	0	0	0	0	0	ND	0	0	0	0	
H5N5	14	0	0	ND	ND	0	0	0	0	0	0	0	ND	0	0	0	0	
H5N5	14	0	0	0	0	0	0	0	0	0	0	0	ND	0	0	0	0	
H5N6	14	0	0	0	0	0	0	0	0	0	0	0	ND	0	0	0	0	
H5N6	14	0	0	0	0	0	0	0	0	0	0	0	ND	0	0	0	ND	
H5N6	14	0	0	0	ND	0	0	0	0	0	0	ND	ND	0	0	0	ND	
H5N6	14	0	0	0	0	0	0	0	0	0	0	0	ND	0	0	0	0	
H5N6	14	0	ND	0	0	0	0	0	0	0	0	0	ND	0	0	0	0	

*dpi, days postinfection; ND, not done; neg., negative; M.O., medulla oblongata; O.E., olfactory epithelium; R.E., respiratory epithelium. The distribution of Influenza A virus matrixprotein was semiquantitatively scored as follows: 0 = no immunoreactivity; 1 = focal/oligofocal immunoreactive cells; 2 = multifocal immunoreactive cells; 3 = coalescing/diffuse immunoreactive cells.

Appendix Table 5. Acknowledgment of laboratories providing sequences in EpiFlu database*

Isolate ID	Country	Isolate name	Submitting Laboratory
EPI_ISL_282143	Italy	A/goose/Italy/17VIR6358–3/2017	Istituto Zooprofilattico Sperimentale Delle Venezie
EPI_ISL_289713	The Netherlands	A/Great Black-backed Gull/Netherlands/1/2017	Erasmus Medical Center
EPI_ISL_282141	Italy	A/swan/Italy/17VIR7064–1/2017	Istituto Zooprofilattico Sperimentale Delle Venezie
EPI_ISL_292223	United Kingdom	A/mute_swan/England/AVP_18_001986/2017	APHA
EPI_ISL_288410	The Netherlands	A/Mute Swan/Netherlands/17017377–001/2017	Wageningen Bioveterinary Research
EPI_ISL_288409	The Netherlands	A/Mute Swan/Netherlands/17017367–012/2017	Wageningen Bioveterinary Research
EPI_ISL_273847	Italy	A/turkey/Italy/17VIR5878–3/2017	Istituto Zooprofilattico Sperimentale Delle Venezie
EPI_ISL_287800	Taiwan	A/spoonbill/Taiwan/DB645/2017	Animal Health Research Institute
EPI_ISL_275433	Russia	A/unknown/Tatarstan/94/2017	State Research Center of Virology and Biotechnology VECTOR
EPI_ISL_275432	Russia	A/unknown/Tatarstan/86/2017	State Research Center of Virology and Biotechnology VECTOR
EPI_ISL_275288	Russia	A/chicken/Tatarstan/88/2017	State Research Center of Virology and Biotechnology VECTOR
EPI_ISL_287907	The Netherlands	A/Duck/Netherlands/17017237–001–005/2017	Wageningen Bioveterinary Research
EPI_ISL_287906	The Netherlands	A/Duck/Netherlands/17017236–001–005/2017	Wageningen Bioveterinary Research
EPI_ISL_292225	United Kingdom	A/canada_goose/England/AV58_18OPpoolEP1/2018	APHA
EPI_ISL_288412	The Netherlands	A/Tufted Duck/Netherlands/17017367–007/2017	Wageningen Bioveterinary Research
EPI_ISL_289714	The Netherlands	A/Black-headed Gull/Netherlands/29/2017	Erasmus Medical Center
EPI_ISL_297235	Russia	A/chicken/Rostov-on-Don/1598/2017	State Research Center of Virology and Biotechnology VECTOR
EPI_ISL_297234	Russia	A/chicken/Rostov-on-Don/1321/2017	State Research Center of Virology and Biotechnology VECTOR
EPI_ISL_291109	Germany	A/common_pochard/Germany-BY/AR09–18-L02421/2017	Friedrich-Loeffler-Institut
EPI_ISL_292224	United Kingdom	A/pochard_duck/England/AVP_18_003254/2018	APHA
EPI_ISL_312376	Georgia	A/Armenian Gull/Republic of Georgia/4/2017	National Center for Disease Control and Public Health, Georgia
EPI_ISL_289713	The Netherlands	A/Great Black-backed Gull/Netherlands/1/2017	Erasmus Medical Center
EPI_ISL_304404	China	A/Fujian-Sanyuan/21099/2017	Fujian Provincial Center for Disease Control and Prevention
EPI_ISL_288437	South Korea	A/duck/Korea/HD1/2017	Animal and Plant Quarantine Agency (O-2144)
EPI_ISL_288436	South Korea	A/mallard/Korea/Jeju-H24/2017	Animal and Plant Quarantine Agency (O-2144)
EPI_ISL_305417	The Netherlands	A/Domestic_Duck/Netherlands/EMC-6/2018	Erasmus Medical Center
EPI_ISL_305416	The Netherlands	A/Domestic_Duck/Netherlands/EMC-2/2018	Erasmus Medical Center
EPI_ISL_303520	Georgia	A/Mallard/Republic of Georgia/1/2018	National Center for Disease Control and Public Health, Georgia
EPI_ISL_268665	The Netherlands	A/Mal/NL-Mastenbroek/16015378–002/2016	Wageningen Bioveterinary Research
EPI_ISL_268669	The Netherlands	A/T_Dk/NL-Monnickendam/16013865–006–008/2016	Wageningen Bioveterinary Research
EPI_ISL_303643	South Korea	A/Mandarin_duck/Korea/K18–3/2018	Avian Diseases Laboratory, College of Veterinary Medicine, Konkuk University
EPI_ISL_303642	South Korea	A/Mandarin_duck/Korea/K17–1896/2017	Avian Diseases Laboratory, College of Veterinary Medicine, Konkuk University
EPI_ISL_303641	South Korea	A/Mandarin_duck/Korea/K17–1894/2017	Avian Diseases Laboratory, College of Veterinary Medicine, Konkuk University
EPI_ISL_303640	South Korea	A/Mandarin_duck/Korea/K17–1893/2017	Avian Diseases Laboratory, College of Veterinary Medicine, Konkuk University
EPI_ISL_303639	South Korea	A/Mandarin_duck/Korea/K17–1891/2017	Avian Diseases Laboratory, College of Veterinary Medicine, Konkuk University
EPI_ISL_303638	South Korea	A/Mandarin_duck/Korea/K17–1889/2017	Avian Diseases Laboratory, College of Veterinary Medicine, Konkuk University
EPI_ISL_303637	South Korea	A/Mandarin_duck/Korea/K17–1887/2017	Avian Diseases Laboratory, College of Veterinary Medicine, Konkuk University

Isolate ID	Country	Isolate name	Submitting Laboratory
EPI_ISL_238148	South Korea	A/Mandarin_duck/Korea/K16–187–3/2016	Avian Diseases Laboratory, College of Veterinary Medicine, Konkuk University
EPI_ISL_288364	Greece	A/chicken/Greece/39_2017b/2017	TVC
EPI_ISL_288363	Greece	A/chicken/Greece/39_2017a/2017	TVC
EPI_ISL_288362	Greece	A/chicken/Greece/39_2017/2017	TVC
EPI_ISL_239393	Japan	A/duck/Aichi/231002/2016	National Institute of Animal Health
EPI_ISL_239416	Japan	A/duck/Fukui/181006/2015	National Institute of Animal Health
EPI_ISL_224753	The Netherlands	A/chicken/Netherlands/15007212/15	Wageningen Bioveterinary Research
EPI_ISL_294779	France	A/duck/France/160051/2016	ANSES
EPI_ISL_294778	France	A/duck/France/160056/2016	ANSES

*ANSES, Agence Nationale de Securite Sanitaire de l'Alimentation, de l'Environnement et du Travail; APHA, Animal and Plant Health Agency; TVC, Thessalonica Veterinary Centre.

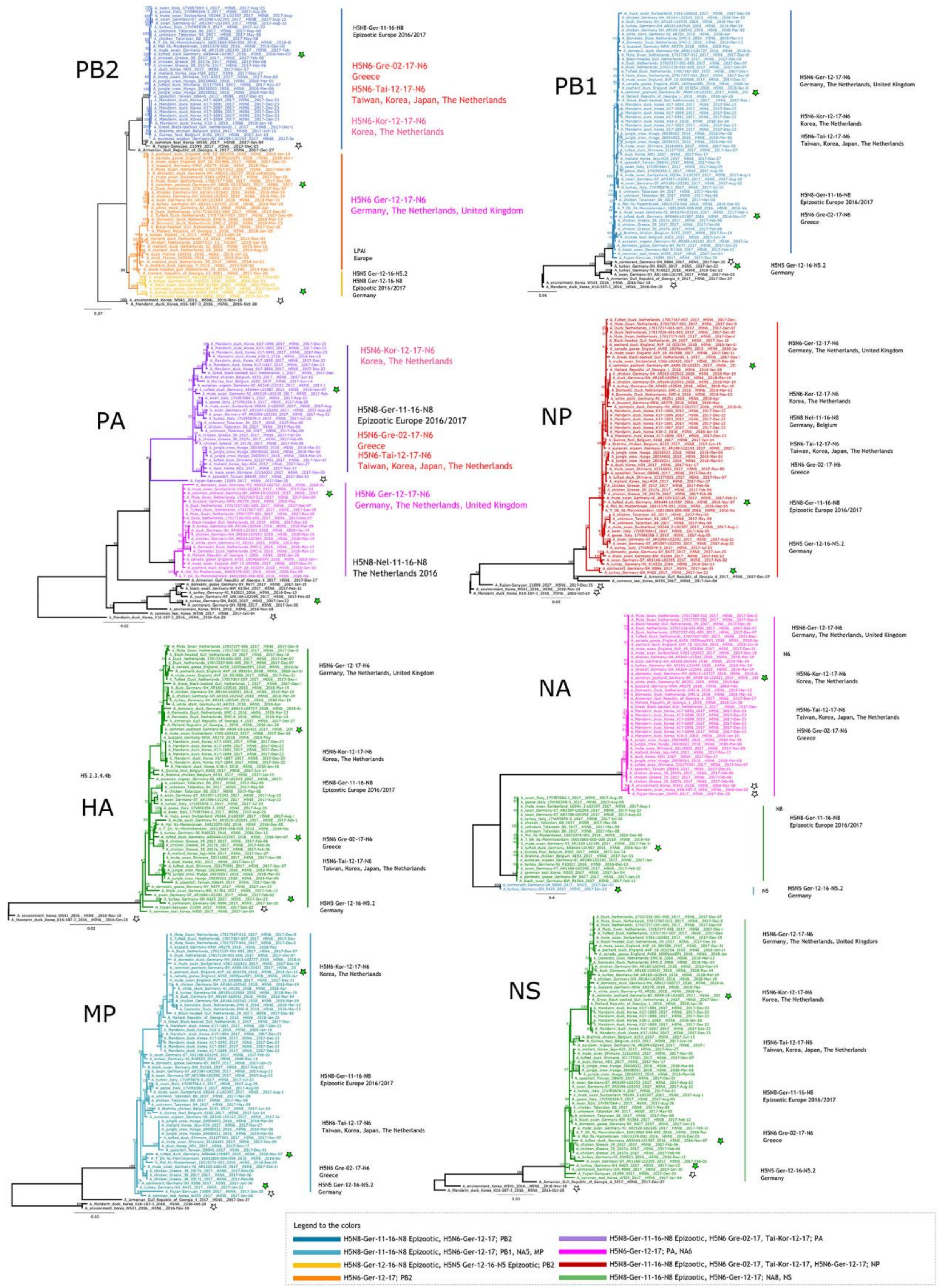

Appendix Figure 1. Phylogenetic analyzes of PB2, PB1, PA, HA, NP, NA, MP, and NS genes from H5Nx viruses done by Maximum Likelihood using RAxML. Bootstrap values of 1,000 cycles >50 are included. Scale bars indicate nucleotide substitutions per site. Reassortants are grouped according to phylogenetic results and shown to the right. Stars indicate strains tested in the ferret animal model described in this and a previous study (green stars), strains tested in different animal models (white

stars), and human strains (white stars). H5N6 viruses analyzed in this study are labeled in different colors according to their clusters (see legend). The reassortants H5N8 and H5N5 Germany are described in detail in (*1*). HA, hemagglutinin; MP, matrix protein; NA, neuraminidase; NP, nucleoprotein; NS, nonstructural protein; PA, polymerase acidic protein; PB1, polymerase basic protein 1; PB2, polymerase basic protein 2.

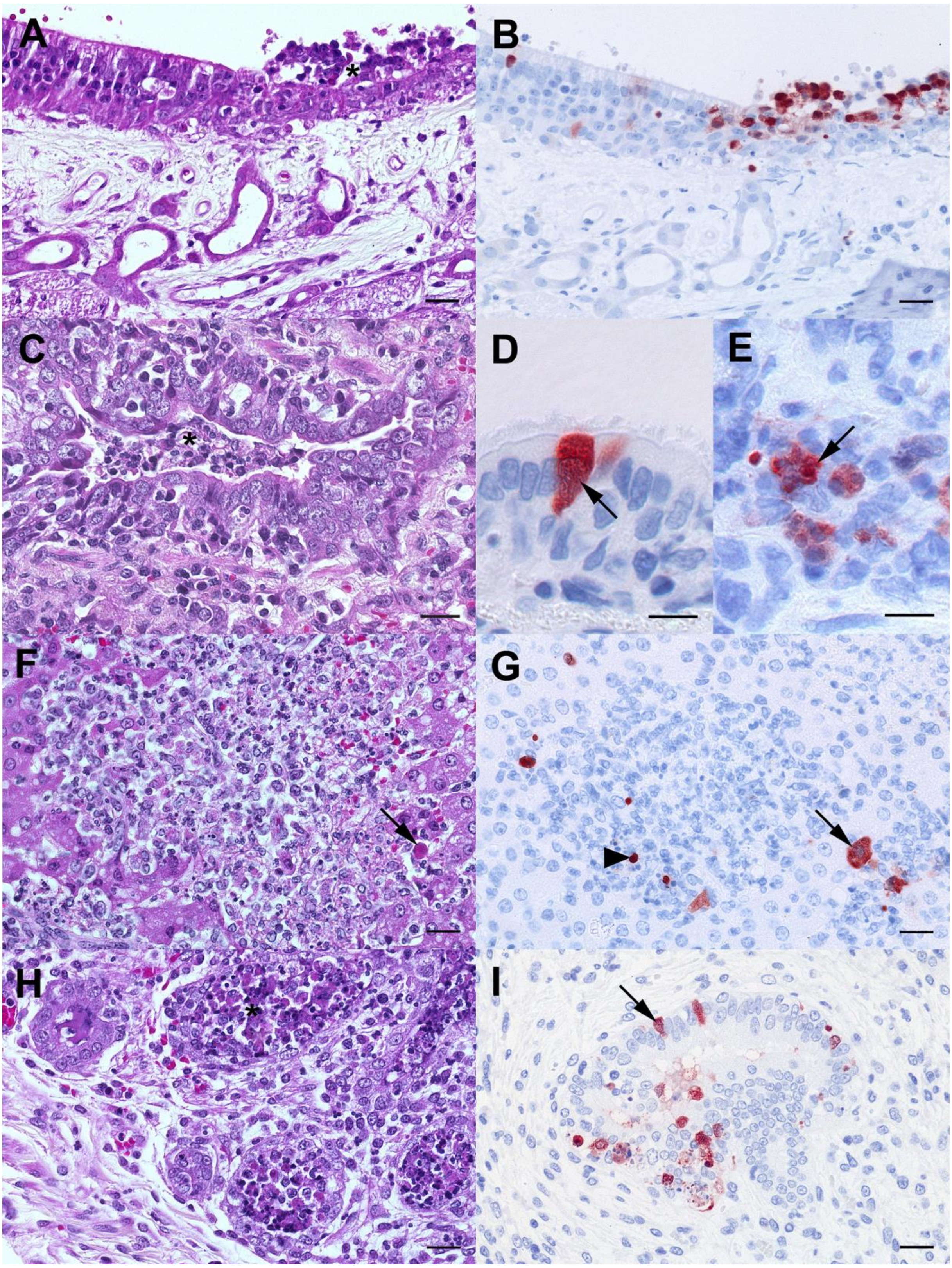

Appendix Figure 2. Histopathological findings in the H5N5-infected ferret necropsied at 7 days postinfection (dpi). A, C, E, G) Hematoxylin eosin. B, D, F, H) Influenza A virus-matrixprotein immunohistochemistry, avidin-biotin-peroxidase complex method, with a monoclonal mouse anti-influenza A virus (strain PR8, A/PR/8/34(H1N1))-matrixprotein immunoglobulin G1 containing hybridoma supernatant (clone M2–1C6–4R3, ATCC HB-64; American Type Culture Collection, Manassas, USA), 3-amino-9-ethyl-carbazol as chromogen and hematoxylin counterstain. A) Nasal

cavity, olfactory mucosa. Mild, focal, subacute, necrotizing rhinitis with degeneration and loss of the olfactory epithelium (asterisk). B) Nasal cavity, olfactory mucosa. Abundant intensely influenza matrixprotein immunoreactive cells and cellular debris within the lesion. C) Lung, bronchioles. Moderate, oligofocal, subacute, necrotizing bronchitis with luminal debris accumulation (asterisk). D) Lung, bronchus. A characteristic bronchial epithelial cell with intense pancellular influenza matrixprotein expression (arrow). E) Lung, bronchiolus. There is intensely influenza matrixprotein immunoreactive cellular debris (arrow) within the lumen of the collapsed and necrotic bronchioles. F) Liver. Moderate, multifocal, subacute, necrotizing hepatitis with infiltrating macrophages, neutrophils, and rare Councilman Corpuscles (arrow). G) Liver. Multifocal intensely influenza matrixprotein immunoreactive intralesional debris (arrow) and fewer hepatocytes (arrowhead). H) Uterine tube. Severe, diffuse, acute, necrotizing salpingitis with nearly complete loss of mucosal epithelium and luminal debris accumulation (asterisk). I) Uterine tube. Multiple influenza matrixprotein immunoreactive epithelial cells (arrow) and debris in luminal recesses in the lamina propria. A–C, F–I) Bar = 20 µm. D, E) Bar = 10 µm.
